# CD11b^+^ interstitial macrophages are required for ischemia‐induced lung angiogenesis

**DOI:** 10.14814/phy2.13721

**Published:** 2018-06-11

**Authors:** Aigul Moldobaeva, Qiong Zhong, Lindsey Eldridge, Elizabeth M. Wagner

**Affiliations:** ^1^ Departments of Medicine and Environmental Health Sciences Johns Hopkins University Baltimore Maryland

**Keywords:** IL‐6, interstitial macrophages, ischemia, MIP‐2*α*, neutrophils

## Abstract

The importance of myeloid cells in promoting neovascularization has been shown in a number of pathological settings in several organs. However, the specific role of macrophages in promoting systemic angiogenesis during pulmonary ischemia is not fully determined. Our past work suggested that cells of monocytic lineage contributed to systemic angiogenesis in the lung since clodronate‐induced depletion of all macrophages resulted in attenuated neovascularization. Our current goals were to define the population of macrophages important for systemic vessel growth into the lung after the onset of pulmonary ischemia in mice. Interstitial macrophages (CD64^+^ MerTK ^+^ CD11b^+^) increased significantly as did the percent of CD45^+^ Ly6G^+^ neutrophils 1 day after the induction of left lung ischemia, despite the fact there was limited cell recruitment due to complete obstruction of the left pulmonary artery in this ischemia model. Since both interstitial macrophages and neutrophils express CD11b, we used CD11b^+^ DTR mice and showed the critical role for these cells since CD11b^+^ depleted mice showed no systemic angiogenesis 7 days after the onset of ischemia when compared to control mice. Coculture of mouse aortic endothelial cells with macrophages showed increased proliferation relative to endothelial cells in culture without inflammatory cells, or pulmonary artery endothelial cells. We conclude that CD11b^+^ leukocytes, trapped within the lung at the onset of ischemia, contribute to growth factor release, and are critical for new blood vessel proliferation.

## Introduction

The importance of inflammatory cells in promoting neovascularization has been shown in a number of pathological settings in several organs. Specifically, macrophages have been shown to be proangiogenic and essential to the overall process of neovascularization. Functioning both in a paracrine manner by providing growth factors, as well as serving as structural components to new sprouting capillaries, recruited macrophages have been confirmed at the site of sprouting blood vessels (Bourghardt Peebo et al. 2011; Avraham‐Davidi et al. 2013; Melgar‐Lesmes and Edelman 2015). The role of macrophages in ischemia‐induced neovascularization has not been fully determined although phenotypically different populations may be involved at different sites within an organ as well as time points in the reparative process (Hakimzadeh et al. 2016). Understanding the normal sequelae and phenotype is important from a tissue engineering perspective to optimize tissue homeostasis (Eaton et al. 2015). However, knowledge of macrophage activation critical for neovascularization in ischemic peripheral organs may not be translatable to the lung. Tissue hypoxia is a key stimulant during ischemia in peripheral organs, which does not occur in the well‐oxygenated environment of the ventilated lung. Previous work from our laboratory showed that myeloid‐derived cells contributed to systemic angiogenesis during pulmonary ischemia since clodronate depletion of all macrophages resulted in attenuated neovascularization (Moldobaeva et al. 2011). Macrophage‐derived cytokines have also been shown to be important for blood vessel growth (Keeley et al. 2008; Hattori et al. 2013). Although several lung cell types are known to secrete growth factors that promote endothelial cell proliferation, macrophage‐derived factors are prominently induced in lung angiogenesis (Srisuma et al. 2003; Keeley et al. 2008). After the onset of chronic pulmonary ischemia, the prototypic M1 macrophage cytokine IL‐6 was shown to be critical for systemic angiogenesis (McClintock and Wagner 2005) as well as the CXC chemokines (Sánchez et al. 2007).

Under normal homeostatic conditions in the lung, interstitial macrophages constitute a very small macrophage subpopulation compared to the more prominent alveolar macrophages. Precursor circulating blood monocytes are recruited to the lung at times of tissue injury (Wilson et al. 2009; Geissmann et al. 2010) where they can both differentiate and proliferate (Jenkins et al. 2011). Yet, it is not clear whether recruited cells are required for the recovery that occurs with ischemia after pulmonary artery obstruction. Since the mouse lacks a subcarinal bronchial vasculature (Mitzner et al. 2000), few inflammatory cells can be recruited to the lung during pulmonary artery obstruction. Thus, the early changes in macrophage phenotype reflect predominantly in situ maturation and/or proliferation.

Our previous work suggested that interstitial macrophages rather than the more abundant alveolar macrophage, were likely important for systemic neovascularization in the ischemic lung (Moldobaeva et al. 2011). Since those observations were reported, however, subsets of lung macrophages have been more extensively characterized (Eldredge et al. 2016; Gibbings et al. 2017; Mould et al. 2017; Reddy and Mehta 2017). Additionally, genetically targeting different macrophage populations in mice is available (Cailhier et al. 2005; Borthwick et al. 2016). Thus, we sought to define the macrophage population most critical for systemic endothelial growth during ischemia. Based on our observations, we sought to further compare the effects of macrophages and neutrophils on the proliferation of pulmonary endothelium versus systemic endothelial cells to better explain the overall process of angiogenesis during lung ischemia. Our work demonstrates a requirement for CD11b^+^ leukocytes for systemic endothelial cell growth in the lung.

## Methods

### Mice

C57BL/6 (male, 6–8 week; Jackson Labs) and CD11b^*DTR*^ (bred on site) mice were housed in a pathogen‐free facility. Experimental protocols were approved by The Johns Hopkins Animal Care and Use Committee (Protocol # MO13M239). Systemic angiogenesis during left lung ischemia was studied in anesthetized (2% isoflurane), ventilated (120 breaths/min, 0.2 mL/breath) mice after left pulmonary artery ligation (Mitzner et al. 2000; McClintock and Wagner 2005; Moldobaeva et al. 2011; Zhong et al. 2016). Using mice that express the human diphtheria toxin (DT) receptor under the control of the CD11b promoter (CD11b^DTR^ mice; Cailhier et al. 2005), the essential nature of CD11b^+^ leukocytes to the process of systemic angiogenesis was studied. In experiments using CD11b^*DTR*^ mice, CD11b^+^ leukocytes were depleted by ip injection of DT (20 ng/g body weight) 1 day before the onset of ischemia.

### Angiogenesis index

Systemic neovascularization of the lung was determined by blood flow assessment 7 days after the onset of left lung ischemia by fluorescent bead (10 *μ*m; Invitrogen) infusion (Zhong et al. 2016). Microspheres lodged in the left lung were quantified following tissue digestion and fluorescent dye extraction. This in vivo approach to the quantification of systemic angiogenesis in the lung has provided a long‐term, consistent, and reproducible assessment of new vessel growth to the murine lung (Mitzner et al. 2000; McClintock and Wagner 2005; Sánchez et al. 2007; Nijmeh et al. 2010; Zhong et al. 2016). Complete validation of this approach to quantify angiogenesis in the lung was previously published (Zhong et al. 2016). Data are presented as % of microspheres in the left lung relative to the total delivered (angiogenesis index).

### Preparation of cell suspensions for FACS

**Figure 1:**
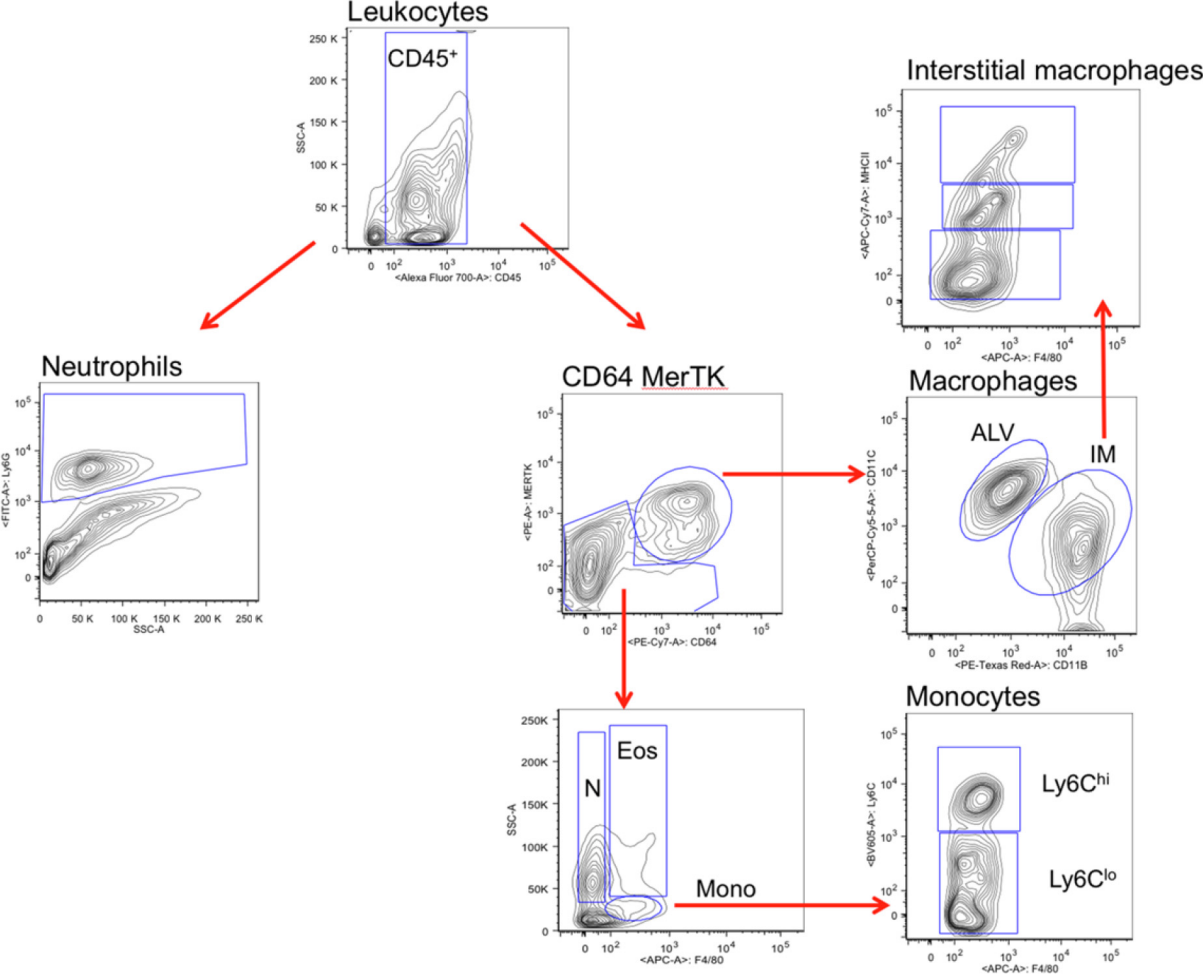
Gating strategy used for CD45^+^ subpopulations. Pregating on LIVE cells, CD45^+^ leukocytes were analyzed for myeloid populations (right fork, Gibbings et al. 2017) as well as neutrophils (left fork; Ly6G^+^ versus side scatter [SSC‐A], Yu et al. 2016), in mouse left lungs D1 after the onset of ischemia. Macrophages (CD64^+^ MerTK ^+^ CD11c^+^), were either alveolar macrophages (ALV; CD11b^−^) or interstitial macrophages (IM; CD11b^+^). Interstitial macrophages (IM) were further evaluated for MHCII ^hi, intermediate, lo^. CD64^−^MerTK ^−^ cells were further defined based on F4/80 staining and side scatter (SSC‐A) with the second analysis of neutrophils (N) as F4/80^lo^ and eosinophils (Eos) as F4/80^intermediate^ and monocytes F4/80^+^. Monocyte subpopulations were either Ly6C^hi^ or Ly6C^lo^. These plots include 65,769 live events.

Using a digestion protocol optimized for leukocyte recovery, left lungs were collected in dissociator tubes (Miltenyi) with Dulbecco's modified Eagle's medium (DMEM) containing Collagenase D (2 mg/mL), 40 U/mL DNase I (Roche Applied Science) and Hepes buffer (10 mmol/L). Tissues were homogenized by a GentleMACS dissociation machine (Miltenyi) and incubated (37°C, 30 min). Samples were passed through a 70 *μ*m nylon strainer (BD Biosciences). Red blood cells were removed using ACK lysis buffer (Invitrogen), and cells were collected and washed thoroughly with cold PBS. Single cell suspensions were labeled in 100 *μ*L of fluorescence‐activated cell sorting (FACS) buffer containing PBS with 0.5% BSA, and incubated for 30 min on ice. The following fluorescence‐conjugated anti‐mouse antibodies were used: CD16/CD32, CD45, Ly6g, and ki67 (BD PharMingen), CD11b, MerTK (eBioscience), F4/80, CD11c, CD64, Ly6c, MHC II and CD31 (Biolegend). Dead cells were excluded, using VIVID (Invitrogen). For each antibody, the fluorochrome and antibody dilution are included in Table 1. Optimal concentrations were determined by antibody titration assay. The gating strategy for defining leukocyte populations is presented in Figure 1.

**Table 1:** List of flurochromes used and antibody dilutions

Antigen	Conjugate	Dilution
CD11C	PerCP‐Cy5.5	1/100
CD11B	PE‐Texas Red	1/200
CD64	PE‐Cy7	1/100
MHCII	APC‐Cy7	1/100
Ly6C	BV605	1/100
Ly6G	FITC	1/100
CD45.2	Alexa Fluor 700	1/100
F4/80	Alexa Fluor 647	1/100
MerTK	PE	1/100
The LIVE/DEAD™ Fixable Blue Stain		1/100

### Flow cytometry

Cell profiles were acquired on an FACS Aria Special Order System (BD), equipped with a four laser‐16 parameter configuration and with laser lines: 1.355 nm/UV‐20mw (Lightwave XCYTE), 2.405 nm/violet ‐50mw (Cube), 3.488 nm/blue ‐100mw (Sapphire 488‐100), 4.635 nm/red ‐30mwv (Cube). The following optical filters and detectors were used to detect each flurochrome in the panel: UV laser detectors: Detector 2: L/D blue; Violet laser detectors: Detector 1: BV421/PacBlue/V450, Detector 2: BV510/PacOrg/V500, Detector 3: BV605; Blue laser detectors: Detector 1: fluorescein isothiocyanate/AF488/green fluorescent protein, Detector 2: PE, Detector 3: PE‐CF594/PE‐TxRed, Detector 4: PerCP‐Cy5.5/PerCP, Detector 5: PE‐Cy7; Red laser: Detector 1: APC/AF647, Detector 2: AF700, Detector 3: APC‐H7/APC‐Cy7. Beads were used to compensate for spectral overlap and software compensation performed. The number of events collected for cells from lung tissue was usually 100,000–20,000 cells, and 20,000 cells from in vitro culture.

Gating controls were fluorescence minus one for each color and to exclude artifacts, time gating, doublets and cell debris exclusion were performed. All data were analyzed with FlowJo 9.9.6 software (Tree Star).

### In vitro macrophage culture

Single‐cell suspensions of left lung (2 × 10^5^ cells/200 *μ*L) were dispensed onto 96‐well plates in DMEM (with 10% fetal calf serum [FCS]). The plate was incubated for 2 h (37°C in 5% CO_2_). Nonadherent cells were discarded, and fresh media was added to the adherent fraction and incubated (37°C in 5% CO_2_). Supernatants were collected after 24 h and used for measuring the production of MIP‐2*α* (CXCL2) and IL‐6 by ELISA (R&D systems). Cell pellets from plates were collected with tissue protein extraction reagent (T‐PER; Thermo Scientific) containing protease inhibitors. Cells were homogenized and total protein measured (BCA kit; ThermoFisher).

### Endothelial cell proliferation

Mouse aortic endothelial cells (Cell Biologics) and pulmonary artery endothelial cells were isolated and cultured using techniques previously established (Moldobaeva et al. 2008). Subsequently, each endothelial cell subtype was seeded on gelatin coated (0.2%) 24 well plates (4 × 10^4^ cells/well) in DMEM with 5% FCS. Inflammatory cells for coculture were isolated from lungs as described above. Cells were purified by magnetic cell sorting using CD45 microbeads and Ly6G‐biotin and F4/80‐biotin antibodies followed with anti‐biotin microbeads (Miltenyi), and according to the manufacturer's protocol. Neutrophils (CD45^+^, Ly6G^+^; >63% purity) or macrophages (CD45^+^, F4/80^+^; >88% purity; 4 × 10^4^ cells/well) were added to the upper chamber of transwells (Corning) and incubated (37°C). After 48 h endothelial cells were washed and stained (CD45^−^, CD31^+^, ki67^+^) for flow cytometry. Endothelial cells with no inflammatory cells added to the upper chamber of transwells served as controls. Supernatants from all the samples were collected and used for measurement of cytokine protein (IL‐6 and MIP‐2) production by ELISA (R&D systems).

### Statistical analysis

Data are presented as the mean ± SE. Analysis of variance was used to compare multiple groups followed by Dunnett's Multiple Comparison test. Two sample comparisons were analyzed with unpaired *t*‐tests. A log transform was performed on cytokine levels from coculture experiments due to the wide concentration range measured; *P* < 0.05 was accepted as significant.

## Results

### Leukocyte characterization after ischemia

Experiments to assess the importance of resident inflammatory cells to the process of neovascularization after the onset of acute ischemia were performed to extend previous observations using specific leukocyte markers in isolated left lung homogenate. The complete gating strategy is presented in Figure 1.

### Macrophages

**Figure 2:**
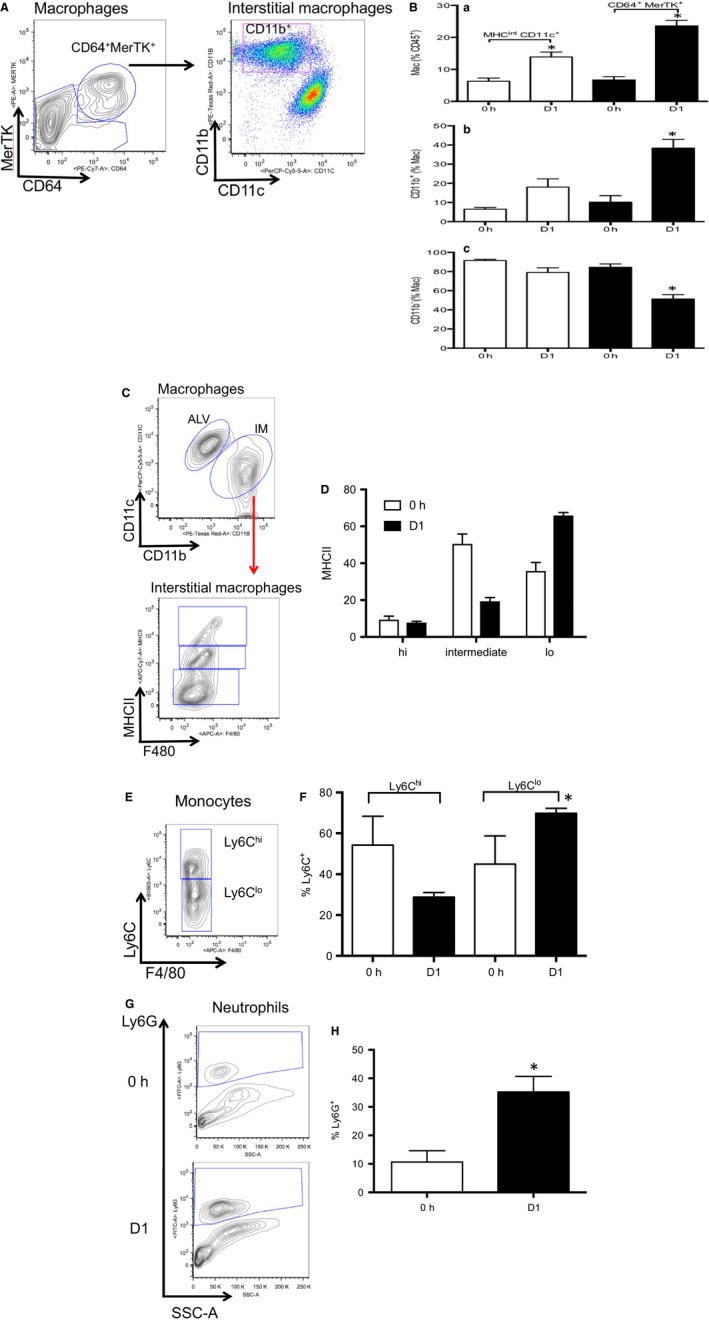
Leukocyte changes in left lung during ischemia. *n* = 4 mice/group, mean ± SE; *P <* *0.05. (A) Representative dot plots showing gates for macrophages (CD64^+^MerTK ^+^ CD11c^+^) and interstitial macrophages (CD11b^+^) in D1 left lung of mice. These plots include 100,976 live events. (B) Average results of macrophage populations 0h and D1 after pulmonary ischemia. Open bars show results using previously used panels to define macrophage populations (Moldobaeva et al. 2011) and black bars demonstrate average values based on gating strategy in (A). A significant increase in the percent of macrophages was observed at D1 (a), which was due exclusively to the significant increase in interstitial macrophages (b), since alveolar macrophages showed a significant decrease (c). (C) Representative contour plots of alveolar macrophages (ALV) compared to interstitial macrophages (IM), and subsequently the activation status of interstitial macrophages (Gibbings et al. 2017). These plots include 10,673 live events. (D) Average results demonstrate that interstitial macrophages were predominantly MHCII ^intermediate^ and MHC ^lo^ both immediately and D1 after ischemia. These cells have been shown to be important for cytokine expression compared to the MHCII ^hi^ phenotype (Gibbings et al. 2017). (E) Representative contour plot of monocytes showing 2 distinct populations of Ly6c^hi^ and Ly6C^lo^. These plots include 1785 live events. (F) Average results demonstrate that initially both Ly6c^hi^ and Ly6C^lo^ phenotypes are equally distributed. However, a shift in monocyte phenotype occurs D1 after ischemia to the more mature Ly6C^lo^ phenotype. (G) Representative contour plots of Ly6G^+^ neutrophils both initially (0h) and D1 after ischemia. A clear increase in the population can be seen in this example. These plots include 72,410 live events at 0h and 52,347 live events at D1. (H) Average results demonstrate a significant increase in the percent of Ly6G^+^ neutrophils D1 after the onset of ischemia.

**Figure 3:**
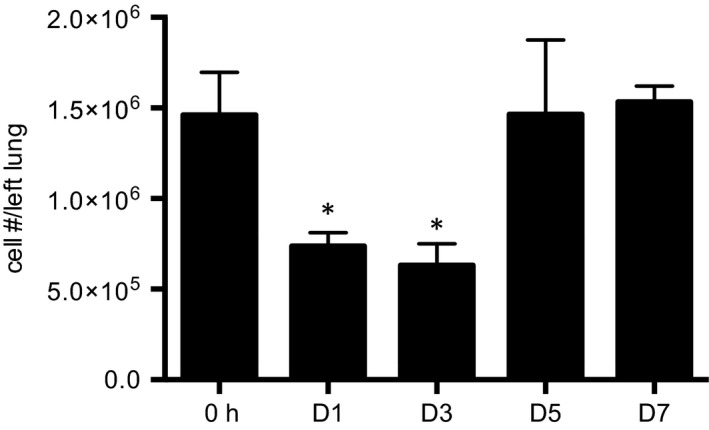
Time course of changes in total leukocyte numbers in dissociated lung. Cell count after left lung dissociation prior to staining procedures demonstrates a significant decrease in cell numbers D1 and D3 after left lung ischemia relative to immediately after the onset of ischemia (0h). *n* = 3–4 mice/group, mean ± SE; *P <* *0.05.

Representative plots from flow cytometry of lung macrophages D1 after the onset of complete left lung ischemia are shown in Figure 2A. We focused primarily on the changes that took place by 24 h after the onset of ischemia (D1) since our previous studies showed maximum changes at this time point (Moldobaeva et al. 2011; Zhong et al. 2016). Quantification of the changes in total macrophages (% of leukocytes), alveolar and interstitial macrophages (% of macrophages) immediately after the onset of ischemia (0h) and D1 after ischemia are presented in Figure 2C (*n* = 4 mice/group). Within the same lung samples, macrophages labeled either as done previously (CD45^+^, CD11c^+^, MHCII^int^; Moldobaeva et al. 2011) or as CD45^+^CD64^+^MerTK^+^ shown in Figure 1, demonstrated an increase in the population of lung leukocytes by D1 compared to what was present at 0h (Fig. 2B, a). This increase was due exclusively to the increased percentage of interstitial macrophages (CD11b^+^; Fig. 2B, b). When this new antibody panel of more selective surface markers was used to identify subpopulations, a significant decrease in the percentage of alveolar macrophages was apparent (CD11b^−^; Fig. 2B, c). Thus, within the same mice, we confirmed our previous observations of the increase in interstitial macrophages using a separate set of surface markers currently used to define this population. In a separate series of mice (*n* = 3–4 mice/time point), the absolute numbers of leukocytes was determined over the early time course after ischemia. A significant decrease in the total number of cells was apparent D1 and D3 after ischemia compared to 0h (Fig. 3). Using the average absolute values as estimates of leukocyte numbers and the confirmed significant increase in the percentage of interstitial macrophages (Table 2), resulted in a 4.5‐fold increase in the calculated number of interstitial macrophages while alveolar macrophages remained the same. Thus, both by percentages or total numbers, a significant increase in interstitial macrophages was seen as a result of left lung ischemia. To evaluate whether macrophage proliferation was responsible for the significant increase in interstitial (CD11b^+^) macrophages at D1, the abundance of the proliferation marker ki67 was determined. Average proliferation of this cell population was 7% and this did not change D1 after the onset of ischemia (*n* = 5 mice/time point; *P* = 0.72).

**Table 2:** Leukocyte populations in left lung tissue homogenate initially (0h) and 1 day (D1) after the onset of left lung ischemia

	% CD45^+^	
	0h	D1
Macrophage	7 ± 1	24 ± 2*
Interstitial	1 ± 1	9 ± 1*
Alveolar	6 ± 1	12 ± 2*
Monocyte	19 ± 4	4 ± 1*
Neutrophil (a)	10 ± 4	33 ± 7*
Neutrophil (b)	11 ± 4	35 ± 5*
Eosinophil	7 ± 1	6 ± 3
*n*	4	4

Data (mean ± SE) are presented as the percent of LIVE CD45^+^ leukocytes. Macrophages and neutrophils increased as a percent of all leukocytes within the lung while monocytes demonstrated a significant decrease. neutrophil (a): Ly6G^+^, neutrophil (b): CD64^−^ MerTK^−^ F4/80^−^.

* *P *<* *0.05.

As seen in Figure 2C and D, further analysis of interstitial macrophages demonstrated a predominance of MHCII^lo^ and MHCII^intermediate^ cells at 0h and D1. Together these two groups comprised the majority, were variable, but have been shown recently to be mature macrophages important for cytokine expression (Gibbings et al. 2017).

### Monocytes

Overall the percent of monocytes as a fraction of all leukocytes decreased significantly (Table 2). Yet the fraction of the more mature Ly6C^+lo^ subtype increased D1 after the onset of ischemia (Fig. 2E and F). These results are consistent with the suggestion that trapped monocytes differentiated to a more mature phenotype (Crane et al. 2014).

### Granulocytes

Neutrophils were determined by two different gating strategies in the present series and were defined by CD45^+^ CD64^−^ MerTK^−^ F4/80^−^ (Gibbings et al. 2017) or by CD45^+^ Ly6G^+^ (Yu et al. 2016, Fig. 1). Both methods demonstrated similar statistically significant increases in the % of neutrophils D1 after ischemia (Table 2; Fig. 2G and H). When cell numbers from the independent series (Fig. 3) were used to estimate total neutrophil numbers based on these percentages, a small increase was observed at D1 relative to 0h (1.6‐fold increase). No change in eosinophils was observed (Table 2).

### Dendritic cells

Dendritic cells (CD45^+^ CD64^+^ MerTK^−^ MHCII^hi^ CD11c^hi^) were <2% of myeloid cells and no changes were seen between 0h and D1 of ischemia.

### CD11b^DTR^ mice

**Figure 4:**
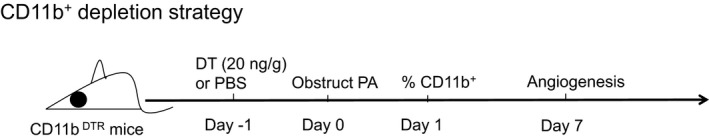
CD11b^+^ depletion strategy and timeline of measurements. CD11b^DTR^ mice were treated with diphtheria toxin (DT) or PBS 24 h prior to the onset of ischemia (Day −1) by pulmonary artery (PA) obstruction. Leukocyte populations were evaluated at D1 and functional angiogenesis was measured 7 days (D7) after the onset of ischemia.

**Figure 5:**
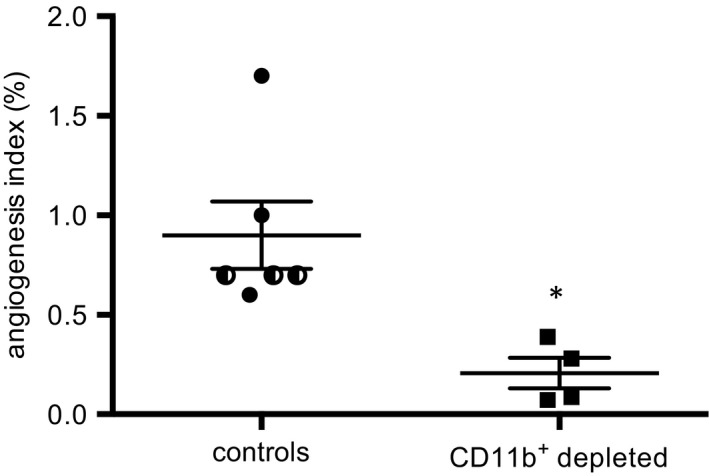
Angiogenesis following CD11b^+^ depletion. The angiogenic index (% microspheres lodged in left lung/total infused) was substantially reduced in CD11b^+^ depleted mice. In control the group, half filled symbols are wild‐type (C57Bl/6) mice treated with diphtheria toxin and filled circles are CD11b^DTR^ mice treated with PBS. *P =* *0.01.

To determine the importance of the CD11b^+^ leukocytes (macrophages, neutrophils, eosinophils) to subsequent angiogenesis, CD11b^DTR^ mice with DT‐induced depletion were studied. Figure 4 demonstrates the CD11b^+^ depletion strategy and timeline of measurements. When this approach was applied, functional angiogenesis was essentially eliminated as shown in Figure 5. We have previously used the 14‐day time point to establish an angiogenic phenotype (Moldobaeva et al. 2011; Zhong et al. 2016). However, given this DT treatment of CD11b^DTR^ mice, a 40% mortality rate was observed thus requiring a shorter time period for the functional angiogenesis evaluation. Control responses included PBS‐treated CD11b^DTR^ mice as well as DT‐treated wild‐type mice as noted by the different symbols used. A significant decrease in the angiogenic index was observed in the CD11b^+^ depleted mice (*n* = 4–6 mice/group; *P* = 0.01). The control response after 7 days of ischemia was 0.9 ± 0.2%, which is similar to what was reported previously (Zhong et al. 2016). This result demonstrates that CD11b^+^ leukocytes are essential to the process of systemic angiogenesis after the induction of pulmonary ischemia.

### Leukocyte changes in CD11b^DTR^ mice

**Figure 6:**
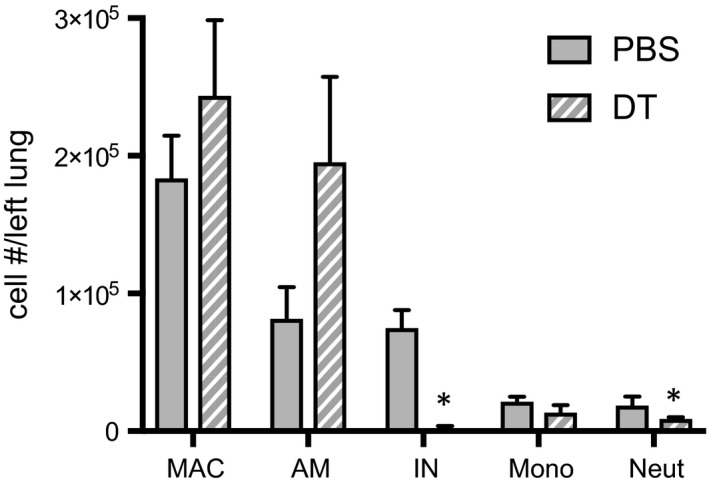
Changes in leukocyte numbers D1 in PBS and diphtheria toxin (DT) treated mice. Cell count after left lung dissociation D1 in PBS (solid) and DT‐treated (striped) CD11b^DTR^ mice. A significant decrease in interstitial macrophages (IN) and neutrophils (Neut) was observed in left mouse lungs after DT treatment. *n* = 3–4 mice/group, mean ± SE; *P <* *0.05.

**Figure 7:**
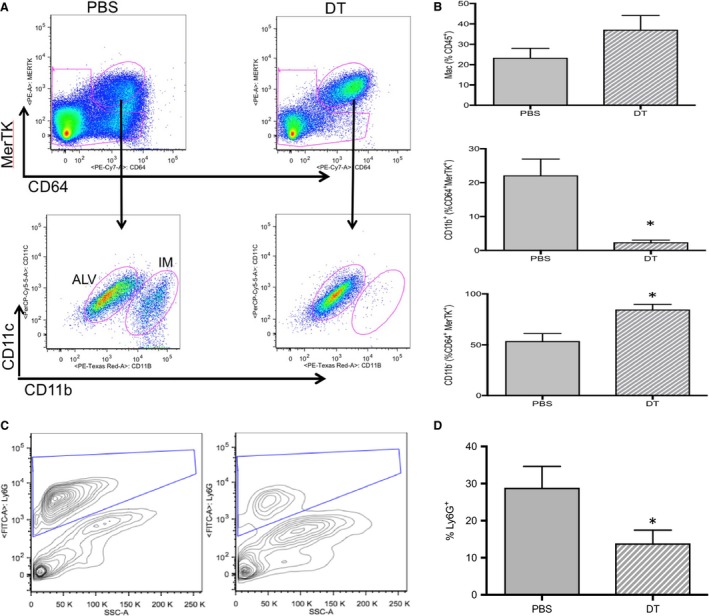
Changes in macrophages and neutrophils D1 in PBS and diphtheria toxin (DT)‐treated mice. *n* = 4 mice/group, mean ± SE; *P <* *0.05. (A) Representative dot plots showing changes in macrophages (upper plots) and interstitial macrophages (IM; lower plots) D1 after the onset of ischemia in PBS‐treated (left plots) and DT‐treated CD11b^DTR^ mice. These plots include 229,019 live events in PBS lungs and 121,320 live events in DT lungs. (B) Average results of macrophage populations D1 after pulmonary ischemia in PBS (solid bars) and DT‐treated (striped bar). A significant decrease in interstitial macrophages (CD11b^+^) was confirmed in DT‐treated mice (b), while alveolar macrophages showed a significant increase (c). (C) Representative contour plots of neutrophils (Ly6G^+^ vs. side scatter [SSC‐A] in PBS and DT‐treated CD11b^DTR^ mice D1 after ischemia). A clear decrease in the neutrophil population can be seen in this example. These plots include 281,400 live events in PBS lungs and 85,293 live events in DT lungs. (D) Average results demonstrate a significant decrease in the percent of Ly6G^+^ neutrophils in DT‐treated CD11b^DTR^ mouse lungs compared to PBS controls, D1 after the onset of ischemia.

To determine the population of CD11b^+^ leukocytes critical to the angiogenic process and to confirm the effectiveness of DT treatment, D1 left lung homogenate of PBS‐treated mice was compared to DT‐treated mice. Average numbers of leukocyte populations are presented in Figure 6, and representative plots from flow cytometry comparing D1 PBS versus D1 DT‐treated mice (Fig. 7A and C) with average data for macrophages and neutrophils compared (Fig. 7B and D; *n* = 4 mice/group). Substantial and significant reductions in CD11b^+^ interstitial macrophages and neutrophils were seen after DT treatment.

### Cytokine release by macrophages

**Figure 8:**
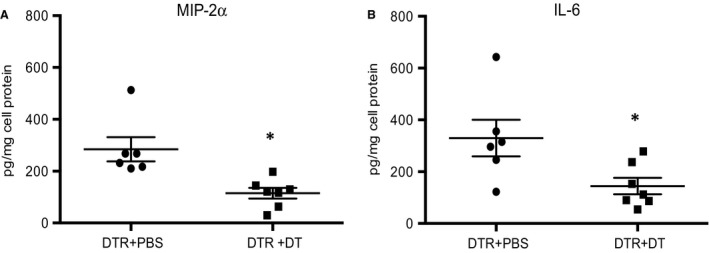
(A and B) MIP‐2*α* and IL‐6 protein secretion are reduced when CD11b^+^ macrophages are depleted. In vitro assay of secreted MIP‐2*α* and IL‐6 protein were both significantly reduced in F480^+^ macrophages isolated from CD11b^DTR^ mice treated with diphtheria toxin (DT) (DTR + DT) compared with CD11b^DTR^ mice treated with PBS (DTR + PBS). Cytokines were normalized for total cell protein (pg/mg cell protein). *P <* *0.05.

To explore one mechanism by which CD11b^+^ macrophages may be contributing to angiogenesis, macrophages were collected from the lungs of PBS and DT‐treated CD11b^DTR^ mice 1 day after the onset of ischemia. Adherent cells were evaluated for their ability to secrete the prototypic M1 cytokine IL‐6 and the prototypic M2 cytokine MIP‐2*α* (CXCL2). Flow cytometric analysis of D1 cells demonstrated that at least 84% of cells recovered were F4/80^+^ macrophages. Figure 8 demonstrates that these macrophages derived from DT‐treated CD11b^DTR^ mice secreted significantly less MIP‐2*α* (*P* = 0.005) and IL‐6 (*P* = 0.028) than macrophages from PBS‐treated CD11b^DTR^ mice (*n* = 6–7 mice/group).

### Endothelial cell proliferation

**Figure 9:**
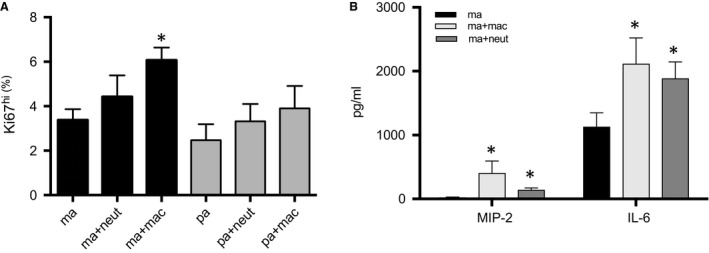
Endothelial cell coculture with isolated lung macrophages or neutrophils. *n* = 5 experiments/group; mean ± SE; *P <* *0.05. (A) Endothelial cell proliferation. Only mouse systemic arterial endothelial cells cocultured with isolated lung macrophages (ma + mac) showed significantly enhanced proliferation (Ki67^hi)^ compared to mouse endothelial cells without leukocytes (ma). Mouse endothelial cells cocultured with lung neutrophils or pulmonary artery endothelial cells with/without inflammatory cells were not different from systemic cells without leukocytes. (B) Cytokines released by macrophages and neutrophils in coculture. Cytokines in supernatants from systemic endothelial cells (ma) cocultured with macrophages (ma + mac) or neutrophils (ma + neut) showed significantly increased levels of MIP‐2*α* and IL‐6.

To examine whether macrophages and neutrophils altered endothelial cell proliferation, in vitro coculture of aortic and pulmonary artery endothelium was studied. As shown in Figure 9A, mouse systemic arterial endothelium (ma) cocultured with isolated lung macrophages (ma + mac) showed the only statistically increased level of proliferation relative to endothelium alone (*n* = 5 exp/group). This was true despite the fact that these were all lung macrophages without CD11b^+^ selection. Although co‐culture with interstitial macrophages was the preferred experiment, it proved technically difficult given the small population of this cell type in normal lungs. No changes in pulmonary endothelial cell proliferation were seen. The cytokines (MIP‐2*α* and IL‐6) that were decreased in isolated macrophages from DT‐treated mice were evaluated in coculture supernatants. Coculture of macrophages and neutrophils both caused a significant increase in MIP‐2*α* and IL‐6, with macrophages showing on average the greatest increase in cytokines (Fig. 9B).

## Discussion

Inflammatory cells are purported to play a key role in promoting systemic angiogenesis in asthma, cystic fibrosis, and supporting primary carcinomas, among several lung diseases. However, identifying which specific cells in these inflammation‐associated pathologies promote neovascularization uniquely, has been difficult to discern. Although mast cells (Mukai et al. 2018) neutrophils (Loffredo et al. 2017), dendritic cells and macrophages (Tavernier et al. 2017), all have been associated with neovascularization and secrete essential growth factors, it has been difficult to separate overlying disease progression with specific cells driving angiogenesis. The goal of this study was to define the population of leukocytes in the lung most important for systemic vessel growth after the onset of pulmonary ischemia in mice. Despite pulmonary artery obstruction, we showed there was a significant increase in the population of interstitial macrophages in the lung at D1 of ischemia. Neither recruitment, due to an obstructed left pulmonary artery, nor proliferation (% Ki67^+^ cells) accounted for the increase in this population of cells, suggesting in situ monocyte differentiation. The number of monocytes decreased with ischemia and those measured were of a more mature Ly6c^lo^ phenotype. When CD11b^+^ interstitial macrophages were depleted in CD11b^DTR^ mice, angiogenesis was essentially eliminated based on the level of systemic perfusion to the lung. Lung macrophages isolated from CD11b^+^‐depleted CD11b^DTR^ mice demonstrated significantly decreased secretion of IL‐6 and MIP‐2*α* compared to controls. Since these two cytokines are known to be critical for angiogenesis in this ischemia model (McClintock and Wagner 2005; Sánchez et al. 2007; Moldobaeva et al. 2010), results are consistent with a paracrine role of CD11b^+^ interstitial macrophages for ischemia‐induced angiogenesis. Furthermore, coculture of macrophages with systemic endothelium but not pulmonary endothelial cells showed enhanced endothelial cell proliferation. This result also emphasizes the proangiogenic nature of the systemic circulation compared to the pulmonary circulation. Our results are consistent with the hypothesis that interstitial macrophages but not alveolar macrophages are critical for new systemic vessel growth in the ischemic lung.

Previous results from our laboratory showed a requirement for macrophages in the lung after the onset of ischemia to promote systemic angiogenesis (Moldobaeva et al. 2011). Macrophage depletion with clodronate liposomes before left pulmonary artery obstruction, reduced systemic neovascularization of the left lung evaluated 14 days after the onset of ischemia. In this study, we used two separate antibody panels and gating strategies to define critical macrophage populations within the same mice. This approach confirmed our past observations (Moldobaeva et al. 2011) and demonstrated a refinement in macrophage phenotyping consistent with current strategies used by others in mouse models of lung disease (Eldredge et al. 2016; Gibbings et al. 2017; Mould et al. 2017; Reddy and Mehta 2017).

Pulmonary artery obstruction stopped pulmonary inflow and prevented recruitment of inflammatory cells. Furthermore, mice have no subcarinal bronchial circulation and systemic arterial inflow from intercostal arteries does not occur until approximately 5 days after the onset of ischemia (Mitzner et al. 2000). Consequently, our results emphasize the importance of in situ monocyte maturation during ischemia‐induced lung injury. Landsman demonstrated that monocyte maturation in the lung to CD11b^+^ macrophages, and subsequently to fully differentiated alveolar macrophages (CD11b^−^), developed along a continuum and were stimulated by the local lung environment (Landsman and Jung 2007; Landsman et al. 2007). With regard to pulmonary ischemia, the Fisher laboratory has shown in a series of studies, the loss of shear stress specifically results in the release of reactive oxygen species (ROS) from pulmonary endothelium (Chatterjee et al. 2008; Browning et al. 2012). In previous studies, our laboratory has confirmed an increase in ROS within the first 24 h after the onset of ischemia and its importance for subsequent systemic angiogenesis (Nijmeh et al. 2010). Whether endothelial cell‐derived ROS can directly activate monocyte differentiation in this model is not clear although intracellular ROS production is required for monocyte differentiation (Zhang et al. 2013).

To fully confirm the importance of the CD11b^+^ leukocyte to the process of systemic angiogenesis to the lung, we used CD11b^DTR^ mice. DT treatment of CD11b^DTR^ mice expressing the human DT receptor under the control of the CD11b promoter, have been used in a wide variety of disease models and have proved to be an effective tool in targeting specifically CD11b^+^ leukocytes (Landsman and Jung 2007; Landsman et al. 2007; Mirza et al. 2009; Borthwick et al. 2016). Depletion of CD11b^+^ leukocytes and confirmed at the D1 time point, had a profound influence on limiting neovascularization to the ischemic left lung. The routinely used endpoint of 14 days to measure the functional perfusion of systemic vessels into the lung in this model could not be used because of the high overall mortality rate. Forty percent of the CD11b^+^ depleted CD11b^DTR^ mice recovered from surgery but died prior to 14 days. Hence, to acquire the data shown in Figure 5, the earlier 7‐day time point was used to measure systemic perfusion to the lung. The control level of angiogenesis was similar to what we showed previously as the angiogenic index at 7 days after ischemia in wild type mice (Zhong et al. 2016) and CD11b^+^ leukocyte depletion significantly reduced the angiogenic index. Although CD11b^+^ leukocytes may perform other essential systemic functions, the results are consistent with the requirement of CD11b^+^ macrophages and possibly neutrophils to promote lung neovascularization by 7–14 days after complete left lung ischemia to preserve life. Our work is consistent with a growing body of literature showing the essential nature of CD11b^+^ leukocytes for angiogenesis. In other systemic organs, current understanding of angiogenesis shows that recruited CD11b^+^ macrophages play a pivotal role in vessel growth in the liver (Melgar‐Lesmes and Edelman 2015), eye (Bourghardt Peebo et al. 2011; Wu et al. 2015), brain (Komohara et al. 2008), heart (Lavine et al. 2014), and several tumor models (Rivera et al. 2015). Interestingly, in a mouse liver regeneration model, infiltrating monocytes were found adjacent to proliferating capillary sprouts while resident macrophages (Kupffer cells), did not interact with vessels. Mice deficient in CD11b^+^ monocytes showed a significant reduction in angiogenesis and liver regeneration after partial hepatectomy. The authors concluded that recruited CD11b^+^ monocytes provided essential proliferative factors (Wnt5a and Ang‐1, and the stalk cell stabilizer Notch1) for blood vessel growth (Melgar‐Lesmes and Edelman 2015). Avraham‐Davidi et al. (2013) confirmed this paracrine function of recruited monocytes in remodeling existing small hepatic vessels. Yet an alternative mechanism has been proposed. CD45^+^ CD11b^+^ myeloid cells were shown to extravasate and form endothelium free tunnels, which over time transformed into perfused capillary sprouts in a corneal inflammation model (Bourghardt Peebo et al. 2011).

The emphasis of this study was on the CD11b^+^ interstitial macrophage, however other leukocytes express CD11b integrin, including neutrophils. After the onset of ischemia, we saw a significant increase in the percentage of neutrophils, which was prevented by DT treatment in the CD11b^DTR^ mice. Whether DT treatment has a direct effect on the depletion of neutrophils or a secondary effect through myeloid cells, appears to be model dependent (Dhaliwal et al. 2012; Borthwick et al. 2016). Our previous work using an antineutrophil antibody demonstrated that neutrophils played little role in angiogenesis in this model (McClintock and Wagner 2005). The importance of neutrophils in the process of angiogenesis in general has been less well‐documented (Seignez and Phillipson 2017). In this study, coculture of systemic endothelial cells showed an enhanced proliferative phenotype only with macrophages. Neutrophils were without effect on endothelial cell proliferation. This result coupled with our previous results lead us to conclude that lung neutrophils do not play the primary role for systemic angiogenesis in this ischemia model. Because they were shown to secrete relevant cytokines, albeit at lower levels, we suggest they provide a secondary source for cytokine growth factors since CD11b^+^ depletion with DT was more effective at blocking angiogenesis than what was shown previously with clodronate treatment.

The precise mechanism whereby CD11b^+^ leukocytes promote systemic neovascularization is not determined. We showed previously that both IL‐6 and the CXC‐chemokines are important for angiogenesis in this ischemia model (McClintock and Wagner 2005; Sánchez et al. 2007). Examination of cytokine gene expression in CD11b^+^ interstitial macrophages was technically difficult given the small number of cells recovered after left lung digestion and sorting. However, in vitro results demonstrated that when CD11b^+^ cells were eliminated from the harvested pool of total lung macrophages using prior DT treatment in vivo, these macrophages secreted significantly less MIP‐2*α* and IL‐6 (Fig. 8). Furthermore, when isolated macrophages were studied in coculture with systemic arterial endothelial cells, a substantial increase in both cytokines was measured in supernatants, perhaps contributing to increased proliferation (Fig. 9). These results are consistent with the importance of CD11b^+^ interstitial macrophages serving a paracrine role in supporting blood vessel growth in the lung.

Two methodological issues require comment. In this study we used labeled microspheres to quantify in vivo the extent of systemic neovascularization in the lung. Histologic evaluation, commonly used in other organs, would require confirming the growth of systemic endothelial networks relative to the pulmonary vasculature and quantification of vessels based on labeling, location, and perfusion patterns. While it is conventional to label endothelium with anti‐CD31, anti‐CD34, or anti‐von Willebrand factor, both systemic and pulmonary endothelial subtypes can take up these labels equivalently as do native compared with angiogenic vessels. Furthermore, lung consolidation due to disease processes can complicate the evaluation of vessel density/unit area. Quantification based on these labels will lead to erroneous conclusions. Similar equivocal results will be obtained if these labels are used for cell sorting or quantification using flow cytometry. Consequently, in this study, we have applied the established in vivo technique using labeled microspheres (Rudolph and Heymann 1967). This approach to the quantification of systemic angiogenesis in the lung has provided a long‐term, consistent, and reproducible assessment of new vessel growth to the murine lung (Mitzner et al. 2000; McClintock and Wagner 2005) and has been validated (Zhong et al. 2016).

Given the model of complete ischemia, both parenchymal and intravascular leukocytes may be important and are included in results. We have focused primarily on the changes of different leukocyte subpopulations as percentages of live cells instead of absolute numbers. We have chosen this approach since in preliminary studies, even slight interexperimental differences lead to different cell viabilities *after* labeling procedures of cell suspensions from dissociated lung tissue. The common technique for live cell quantification using trypan blue exclusion is typically performed *prior* to labeling. Hence in our hands, this approach to cell count provides an overestimation of actual cell viability, which is amplified in subsequent calculations of cell number. For these reasons we believe the leukocyte subpopulations described as percentages of live cells recovered, provide the most accurate representation of results.

In summary, we have shown that despite pulmonary artery obstruction, there was a significant increase in CD11b^+^ interstitial macrophages in the lung D1 after the onset of ischemia. Neither recruitment nor proliferation accounted for the increase in this population of cells, suggesting in situ monocyte differentiation to the more mature interstitial macrophage. When CD11b^+^ cells were depleted in CD11b^DTR^ mice, angiogenesis was prevented. Secretion of IL‐6 and MIP‐2*α* from total lung macrophages was reduced when the CD11b^+^ subpopulation was depleted. We conclude that these two proangiogenic cytokines from CD11b^+^ leukocytes serve an essential paracrine role in ischemia‐induced angiogenesis in the lung.

## Conflict of Interest

None declared.
